# A Case of Isolated Gastrointestinal Histoplasmosis

**DOI:** 10.7759/cureus.2951

**Published:** 2018-07-09

**Authors:** Jasmine Bhinder, Amit Mori, Wenqing Cao, Anju Malieckal

**Affiliations:** 1 Gastroenterology, The Brooklyn Hospital Center/Affiliate of the Mount Sinai Hospital, New York, USA; 2 Pathology, NYU Langone Hospital, New York, USA; 3 Gastroenterology, NYU Langone Hospital, New York , USA

**Keywords:** intestinal histoplasmosis, disseminated histoplasmosis, acquired immunodeficiency syndrome

## Abstract

Histoplasmosis is a self-limited and asymptomatic disease in immunocompetent individuals. Patients with untreated human immunodeficiency virus (HIV) or immune suppression due to medications such as corticosteroids can present with disseminated and life-threatening infections. We present a case of a 60-year-old female that presented with recurrent diarrhea that was found to have isolated gastrointestinal (GI) histoplasmosis. The rarity of this case is due to the isolated colonic involvement and lack of respiratory symptoms (the portal of infection). In conclusion, clinicians should be aware of isolated histoplasmosis affecting the GI tract and careful endoscopic evaluation with adequate sampling is warranted to confirm the diagnosis.

## Introduction

Histoplasma capsulatum is a dimorphic fungus, meaning it grows as a mold in the environment and as yeast at temperatures greater than 37°C. It can be found worldwide, but particularly in North and Central America. Within the United States, it is most common in Midwestern states along the Ohio and Mississippi River valley [1].

Histoplasmosis primarily presents as either a self-limited respiratory infection or is asymptomatic. However, in immunocompromised states such as those with human immunodeficiency virus (HIV) infection and/or acquired immune deficiency syndrome (AIDS), patients can present with disseminated infections involving multiple organ systems [2-3]. Here we present a case of disseminated histoplasmosis in an elderly female. The rarity of this case is due to the isolated colonic involvement and lack of respiratory symptoms (the portal of infection). In addition, this was the inciting event, which led to a diagnosis of AIDS in this patient.

## Case presentation

A 60-year-old female presented with three days of diarrhea and diffuse abdominal discomfort. She reported having five to six bowel movements for two days followed by an additional 15 bowel movements prior to admission. She described that her bowel movements were watery and yellow in appearance. The patient also complained of generalized myalgias and subjective fevers intermittently. She denied any hematochezia, melena, or recent weight loss.

She was hospitalized three weeks prior to this admission with similar symptoms. During that time, she was found to have mild colitis and workup including gastrointestinal (GI) polymerase chain reaction (PCR), stool ova and parasites, Clostridium difficile (C.diff) testing by PCR were all negative. The patient was started on a 10-day course of ciprofloxacin and flagyl. The patient stated that diarrhea resolved with the antibiotics, but restarted two days after completion.

**Figure 1:**
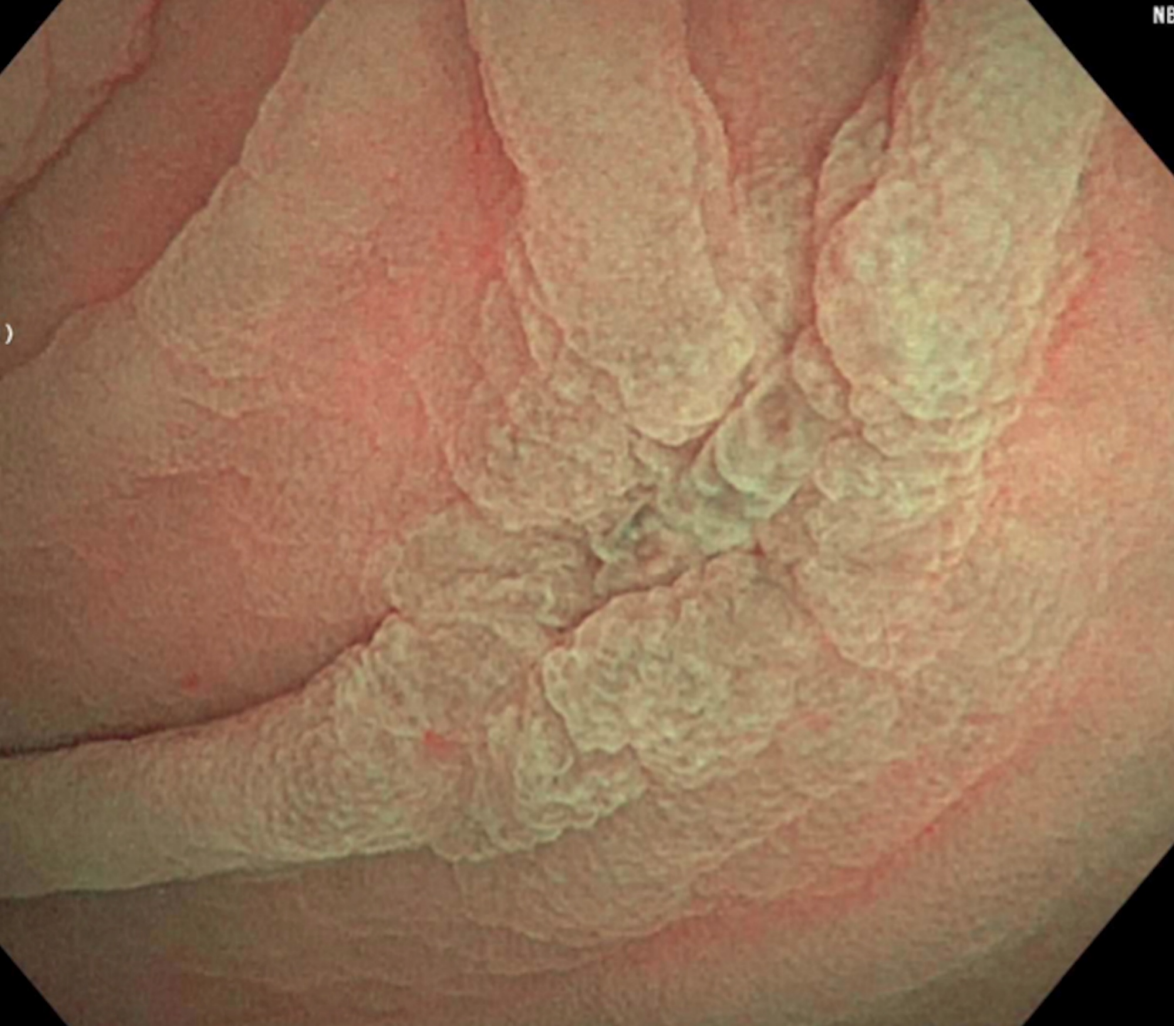
Endoscopy - Isolated 5 mm ulcer in the cecum.

**Figure 2:**
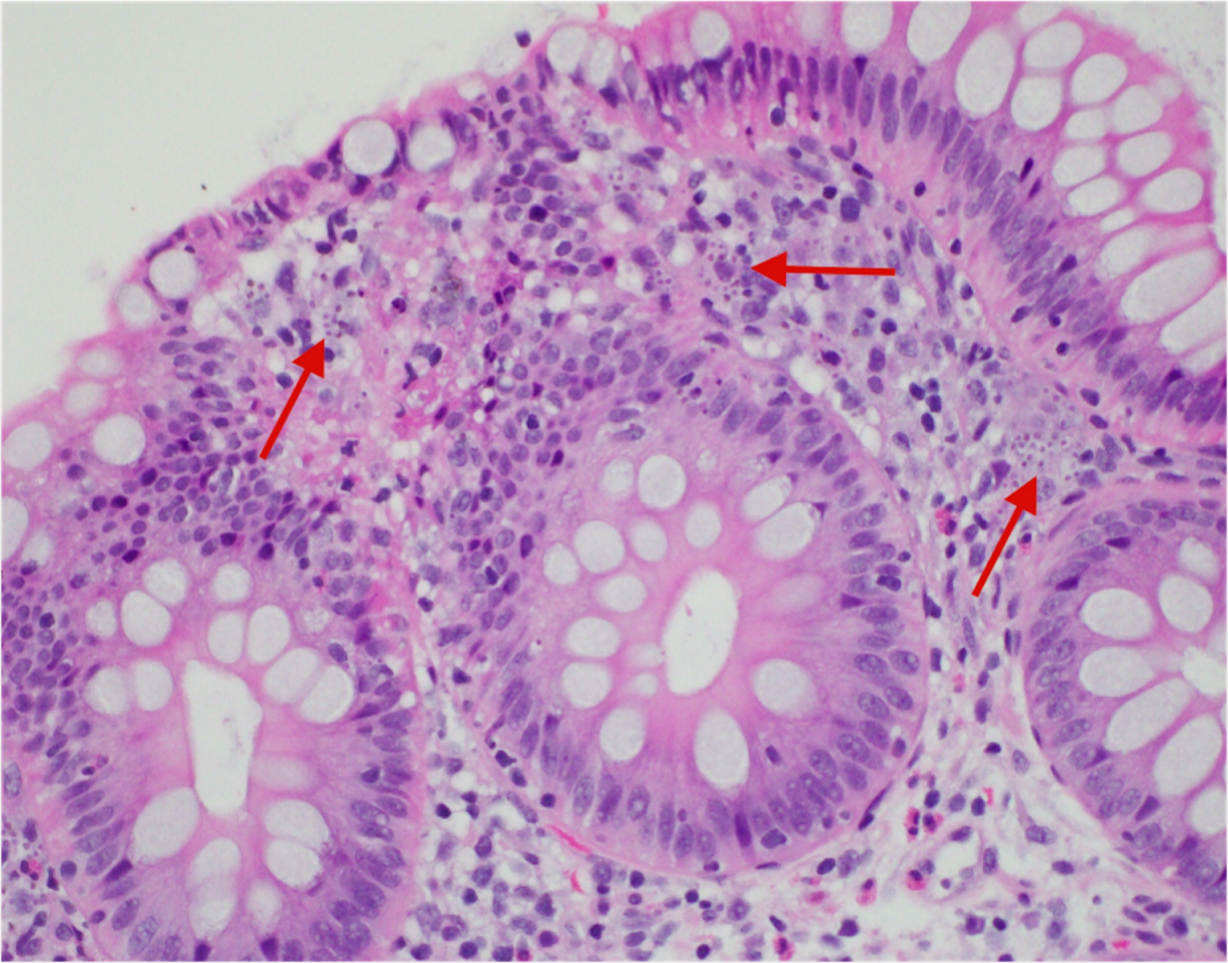
Histiocytosis with intracellular microorganisms within the lamina propria.

**Figure 3:**
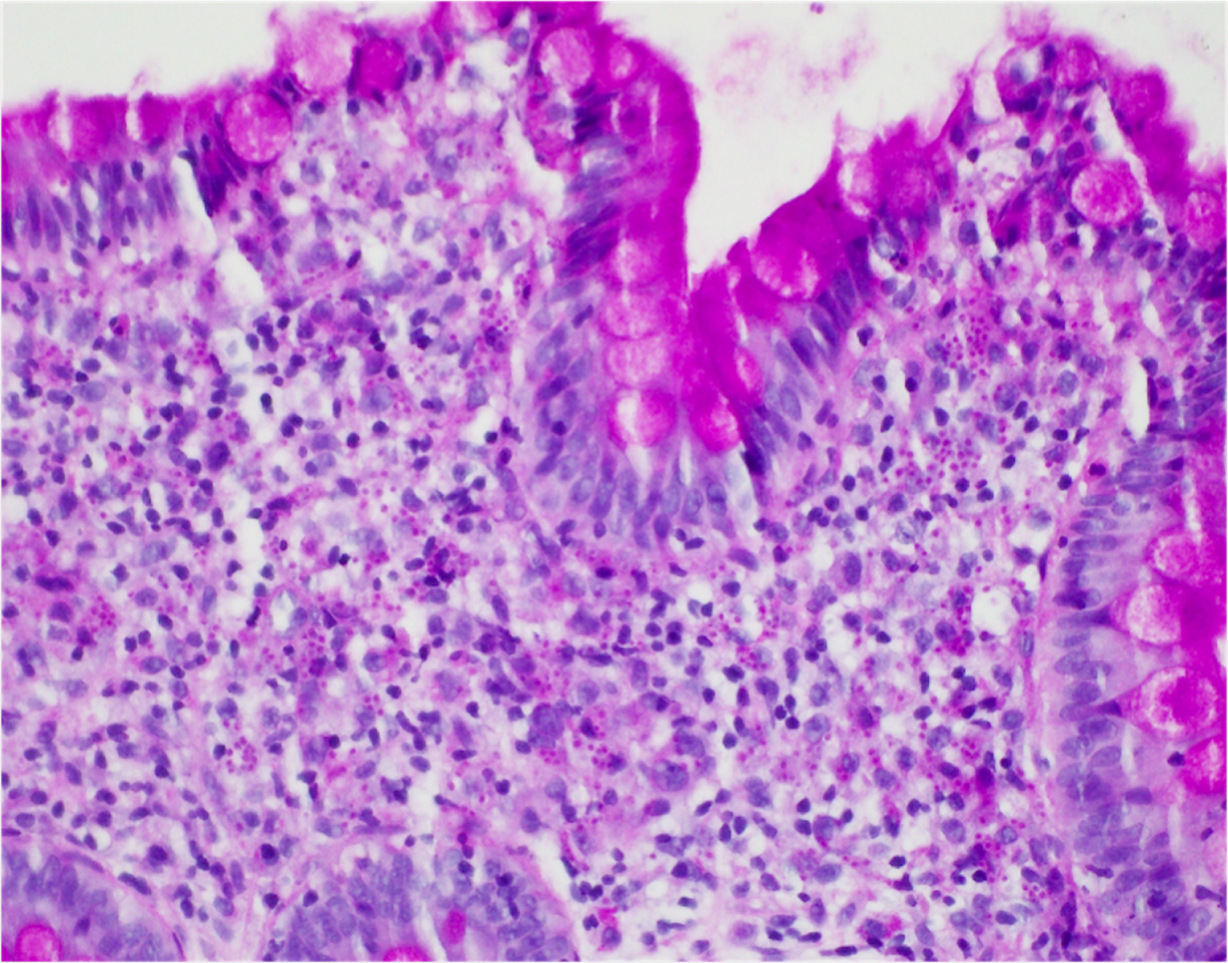
Positive periodic acid Schiff stain.

**Figure 4:**
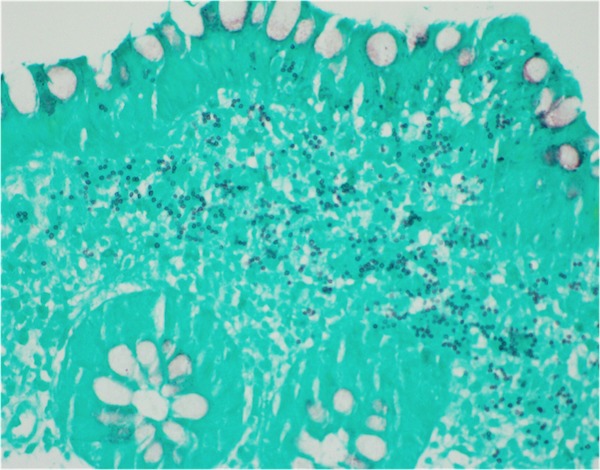
Positive Grocott's methenamine silver stain.

During admission, the patient was started on intravenous (IV) fluids and stool samples were collected and sent to the lab. Stool PCR, C.diff, cultures and ova/parasite testing all again came back negative for the second time. Subsequently, a colonoscopy was performed that revealed a solitary five-millimeter ulcer in the cecum (Figure 1). Biopsies were taken with cold forceps and histopathological analysis confirmed lamina propria histiocytosis with intracellular microorganisms consistent with histoplasmosis (Figure 2). Grocott’s methenamine silver stain and Period acid-Schiff stain were both positive, further confirming the diagnosis of histoplasmosis (Figures 3-4). Subsequently, the patient tested positive for HIV with a CD4 count of 59 and viral load of 140,000. The patient was started on IV amphotericin B with acetaminophen and diphenhydramine premedication. She was also started on a combination of abacavir, dolutegravir and lamivudine daily for HIV infection, sulfamethoxazole/trimethoprim for pneumocystis jiroveci prophylaxis and nystatin for oral thrush. She continued to improve clinically in the hospital and was then discharged home on IV infusion to receive the last three days of amphotericin B to complete a total of 14 days. The patient was then switched to oral itraconazole and scheduled to follow up with an infectious disease specialist.

## Discussion

Histoplasma capsulatum is a dimorphic fungus that primarily presents as a self-limited respiratory illness in immunocompetent individuals. When inhaled into the lungs, it is actively cleared in patients with an intact cell-mediated immune response [4]. T-cell activated macrophages will engulf the organism and clear the yeast cells, leaving the patient with mild or no symptoms at all. While the majority of patients are asymptomatic, those with immunocompromised states can present with disseminated and life-threatening infections [4]. Patients with HIV, on chronic corticosteroids or biological agents, such as tumor necrosis factor-alpha inhibitors, are at high risk of progressive infection. This is primarily due to the lack of macrophage activation from helper T cells. The yeast cells remain engorged within the macrophages and spread via the lymphatics and blood throughout the reticuloendothelial system. Therefore, the most common extra-pulmonary sites involved include the lymph nodes, liver, spleen and bone marrow [4].

Approximately 10% of patients with disseminated histoplasmosis (DIH) present with GI symptoms. However, during autopsy, about 70% of patients with DIH have GI involvement [5-6]. Lesions within the GI tract include polyploid masses and ulcerations and they are most commonly present within the ileum and colon. Due to these characteristics, they can easily be mistaken for Crohn’s disease or ulcerative colitis. Starting immunosuppressive therapy such as steroids or TNF-alpha inhibitors can lead to disastrous results and further progression of histoplasmosis if it is not ruled out before [7]. Therefore, during colonoscopy high degree of clinical suspicion required to avoid missing the diagnosis of rare conditions such as this one, especially prior to starting immunosuppressive therapy.

DIH has a good prognosis with the appropriate antifungal treatment. The options for treatment primarily include a lipid formulation of amphotericin B and itraconazole [8]. Amphotericin B is recommended for initial therapy in patients who are significantly ill and require hospitalization. This is due to the fact that it eradicates fungemia more effectively and quickly compared to itraconazole. Itraconazole is recommended in patients with mild or moderate symptoms and used as step-down therapy after initial treatment with amphotericin B [8]. According to the Infectious Diseases Society of America, clinical practice guidelines for DIH without CNS involvement recommend liposomal amphotericin B (3.0 mg/kg daily) for one to two weeks, followed by oral itraconazole (200 mg three times daily for three days and then 200 mg twice daily for a total of at least 12 months) [9].

## Conclusions

Isolated histoplasmosis without any respiratory symptoms is very rare. In patients with disseminated histoplasmosis, GI symptoms are only found in 10% of cases, however, in this case presentation, the patient presented with recurrent diarrhea. In conclusion, clinicians should be aware of isolated histoplasmosis affecting the GI tract aside from pulmonary involvement and a thorough history and biopsy during endoscopic evaluation should be obtained to confirm the diagnosis.
